# The Social Return on Investment in programs designed for young people living with a family member who experiences mental health challenges: study protocol

**DOI:** 10.3389/fpubh.2024.1411580

**Published:** 2025-01-07

**Authors:** Andrea Erika Reupert, Nerelie Claire Freeman, Nivedita Nandakumar, Rochelle Hine, Rebecca Cain, Kim Foster

**Affiliations:** ^1^School of Educational Psychology and Counselling, Faculty of Education, Monash University, Clayton, VIC, Australia; ^2^School of Rural Health, Faculty of Medicine, Nursing & Health Sciences, Monash University, Clayton, VIC, Australia; ^3^Independent Researcher, Melbourne, VIC, Australia; ^4^School of Nursing, Midwifery and Paramedicine, Faculty of Health Sciences, Australian Catholic University, Melbourne, VIC, Australia

**Keywords:** social return on investment, social cost–benefit analysis, young carer, children, parental mental illness

## Abstract

The purpose of this paper is to describe the protocol for the evaluation of programs offered by the Satellite Foundation, designed for, and with, children and young people aged between 8 and 25 years who have family members experiencing mental health challenges. To achieve this, the Social Return on Investment (SROI) method was chosen. SROI is an economic measurement tool used to apply a monetary value to socially situated outcomes. In this study, SROI will be used to provide a means of quantifying the social impact generated by various programs offered by the Satellite Foundation, a community-based mental health organisation. These programs are designed for children and young people who have a family member who experiences mental health challenges, with the aim to promote resilience, hope and connectedness. Given that traditional financial metrics often fail to capture societal benefits, SROI offers a systematic approach to measuring the economic and often intangible social outcomes of any given endeavour. This protocol will describe the SROI method, who the stakeholders are, and how they are engaged. The rationale for the monetisation of outcomes is shown. Other SROI steps are presented, including how impact was established, and the proposed method of calculating the SROI. The limitations and potential benefits of this economic measurement approach are also discussed.

## Introduction

Family systems theory posits that the health of one member of a family can have a profound impact on the well-being of other family members (1). Likewise, young people who have a parent, sibling or other family member who experiences mental health challenges confront complex issues that can influence their own mental health, developmental and/or behavioural outcomes. Many of these young people assume caring responsibilities in their families (2), a role which can provide young people a sense of purpose and strengthen family bonds, but if onerous, can adversely impact their wellbeing and friendship groups (3). Similarly, while many children and young people in these families identify independence and compassion as positive outcomes (4), others report negative outcomes including low academic attainment (2, 5) and their own mental health and adjustment issues (6). These young people are often considered to be “invisible” as services focus on the needs of their family member and typically do not intervene with children unless there is evidence of abuse or neglect (7). However, there is much that can be done to mitigate these potential negative impacts, if given the necessary support.

There are several programs that have been designed for this population group. Some programs target children aged 8–12 (8) and others assume a whole-of-family approach (9). Some are offered online (10) and others are offered as residential camps (11). The target group and content (e.g., psycho-educational, cognitive behavioural) varies and is often determined by organisational remit (12). Programs for children and young people are typically based on peer support and aim to offer respite from caring responsibilities, and promote connectedness, adaptive coping skills, emotional regulation and mental health literacy (13). Psychoeducation is a common intervention ingredient as some young carers do not have an accurate knowledge about mental illness prognosis and treatment (14). Cognitive behavioural approaches are often used to promote adaptive coping, regulate emotions and build resilience for this target group (15). Overall, such programs report positive outcomes, with a systematic review and meta-analysis finding a significant reduction of the incidence of mental illness in children and young people, and a reduction of internalizing symptoms in the year following the intervention (16). However, another systematic review found only two interventions measured the future risk of developing a mental illness (17). Though similar to other reviews, at post intervention, children reported a significant decrease in internationalising symptomatology. There is a scarcity of longitudinal studies that follow children over an extended period, with many using qualitative evaluations or relying on evaluations that assess at baseline and then immediately post intervention (12). In addition, knowledge gaps exist around demonstrating whether programs for these young people provide value for money.

There have been some, albeit few, economic evaluations conducted on interventions for young people living in families where a member experiences mental health challenges. Wansink et al. (18) assessed the cost-effectiveness of a preventive care-management program for families with a parent with mental illness from a health care, social care and societal perspective. They found the program to be costlier but more effective than treatment-as-usual. Another program focused on children and mothers, where both family members had anxiety and all children received Cognitive Behaviour Therapy (CBT) (19). Creswell et al. (19) were specifically interested in the cost-effectiveness of two additional interventions, where one group of mothers was provided with CBT, while the other group was provided with a program designed to target anxiogenic features of the mother–child relationship. They found positive outcomes for children across all treatment arms, though neither adding CBT for mothers, nor focusing on the parent–child relationship, conferred significant benefits to children or mothers. In terms of their economic evaluation, they concluded that focusing on the child–mother dyad, rather than providing mothers with CBT alone, may be a cost-effective psychological approach for the treatment of child anxiety problems in the context of maternal anxiety disorders. Finally, in Germany, Waldmann et al. (20) attempted to establish the cost utility of an eight-week program for families where a parent has a mental illness but did not find significant differences in resource use, costs or cost utility between the intervention group of families and families receiving ‘treatment as usual’.

The insights gained from economic evaluations can contribute to evidence-based decision-making, enhanced accountability, and the optimisation of outcomes relative to costs. However, as argued by Corvo et al. (21), value is not defined by economics alone; value must incorporate social and/or environment components. Corvo et al. (21) emphasised that “while economic value is created when there is a financial return on an investment, social value is produced when people’s lives are improved owing to the successful combination of resources, input and processes” (p. 49). The combination of these components has led to a number of methodologies for assessing the economic but also the social value of programs (21).

This paper presents a protocol for a Social Return on Investment (SROI) methodology for programs designed for, and with, young people living with a family member who experiences mental health challenges. Based on a long-established clinical history and drawing on other similar program evaluations conducted over several years (8, 10, 11, 22), Satellite’s programs are designed to mitigate the risks associated with having a family member who has a mental illness by promoting children’s wellbeing, connectedness, adaptive coping, a sense of hope and resilience. In various mediums and targeting different age groups, Satellite provides various programs, using creativity and informal psychoeducation, alongside opportunities for young carers to connect with others who share similar life experiences. A SROI approach was chosen to extend the current research base by providing a comprehensive evaluation of impact beyond traditional financial metrics. Programs for these young people often yield intangible benefits such as adaptive coping and improved resilience, and the SROI methodology has the potential to quantify these benefits, providing a clear picture of its true value (23). By providing an account of the social and economic value generated, SROI can assist in the future planning of policymakers, funder and managers. It was for these reasons that the SROI method was chosen.

### Social Return on Investment approach

Social Return on Investment (SROI) is one of the most well-known social impact methods (21) and has been regarded as the “nearest to a current industry standard for project or organisational level social impact reporting” [(24), p. 21]. SROI is an economic measurement tool used to apply a monetary value to socially situated outcomes (25, 26). It seeks to establish how inputs (e.g., staffing) are converted to outputs (including the activities undertaken to deliver the outcomes) and subsequent participant outcomes (e.g., an aspect of improved quality of life such as self-esteem or mental health).

A core feature of SROI methodology is the engagement of stakeholders to determine which outcomes are relevant and deemed to be most important. Another key feature of SROI methodology is to assign monetary values to program outcomes, which may not have market prices (27). As SROI seeks to monetize non-financial factors, there is a need to identify financial proxies that can be used to estimate the positive (or negative) social value created by participating in a given program (25, 26). Ultimately, SROI results in a ratio, such as 3:1, which in this instance shows that for every dollar invested in a program (or organisation), a social value of three dollars is created. The final SROI ratio is not intended to indicate financial value but instead conveys a social value currency (28).

SROI studies can play an important role in how social enterprises conceptualise, measure and communicate their achievements (23) and may be used by governments and philanthropists when making funding decisions (29). The SROI process can help organisations better understand the processes that impact their stakeholders by identifying the links between activities and impacts. As a relatively new methodology, it is important to be transparent about the SROI methodology applied, especially when developing some of the more ambiguous and challenging steps involved in the methodology, including measurement indicators and the proportion of the outcome that may have occurred without any intervention having occurred (28).

**Figure 1:**
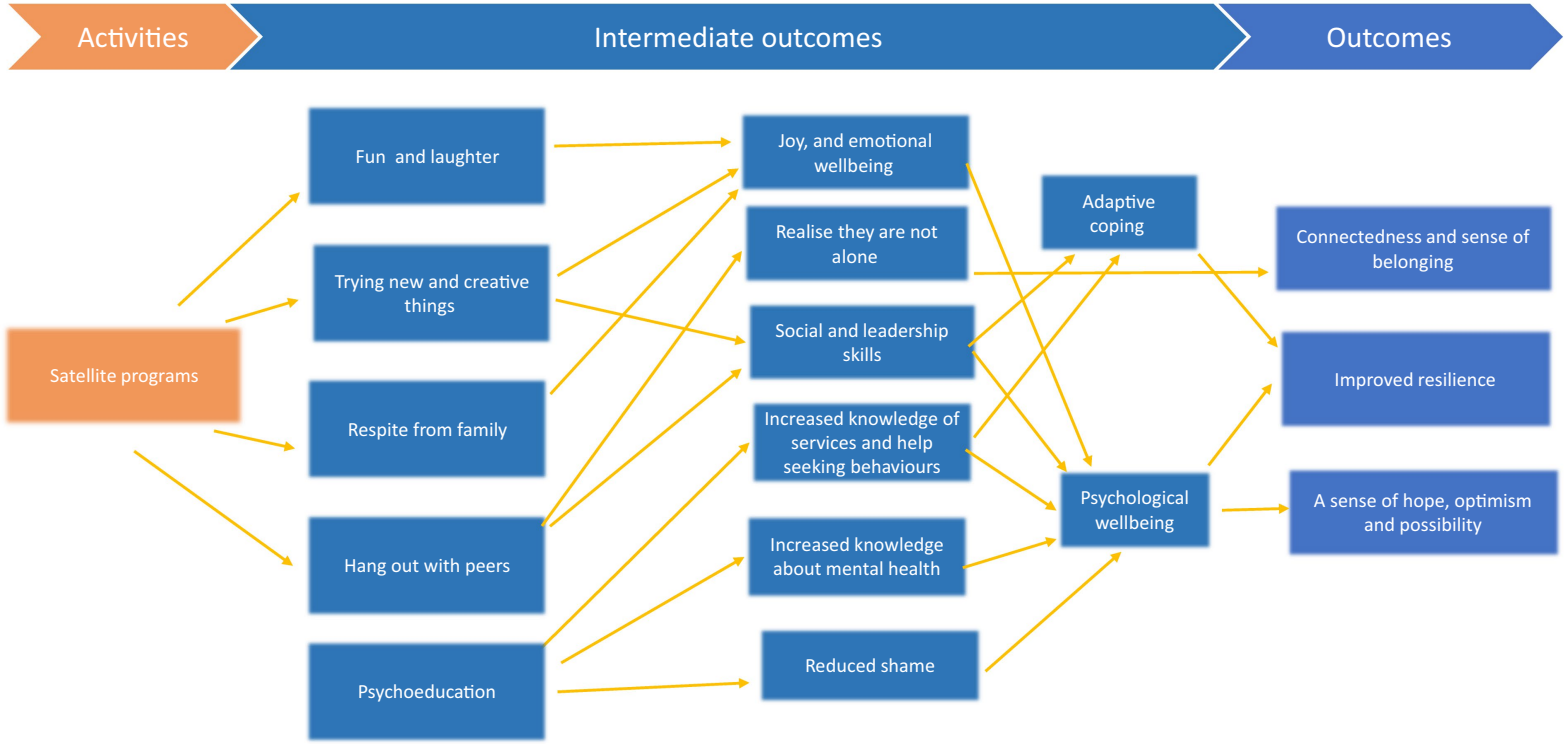
Theory of change.

The purpose of this paper is to describe the SROI protocol for the evaluation of programs designed for, and with, children and young people aged 8–25 years, and who have family members (parents/guardians and/or siblings) who experience mental health challenges. The programs are offered by the Satellite Foundation (hereafter Satellite), an Australian not-for-profit organisation. Satellite provides various in-person and online programs for these children and young people, with the aim of promoting connectedness, wellbeing, and resilience. Programs vary in *length*, *approach* (for example some involve creative activities, others are more psychoeducational) and *medium* (online and face-to-face) and target different *age* groups. Participants can choose any number of programs to join and may participate in multiple programs. While it is acknowledged that the three identified programs have varying lengths, approaches and mediums, they share the same Theory of Change (Figure 1). As Arvidson et al. (23) argued, a consistent Theory of Change allows for a structured approach for evaluating outcomes across different programs. Moreover, the mixed method approach employed ensures that the nuances of each program are captured while maintaining a consistent framework for evaluation (26). Satellite actively promotes and encourages young people to stay engaged via their connecting procedures where they maintain contact between programs. The SROI for Satellite is part of a larger evaluation currently underway (30).

Protocol papers enhance research transparency, prevent unnecessary duplication of research and can provide a useful guide for subsequent research to be undertaken (31). Nicholls et al. (26) indicated that SROI studies can focus on two distinctively different aims, either evaluative or forecast. An *evaluative* SROI is conducted retrospectively on outcomes that have already occurred and the financial costs associated with obtaining those outcomes. A *forecast* SROI aims to predict potential financial costs based on the social value, if the programs meet their intended outcomes. Given the study is still underway, with data still being collected, this current project is a forecast SROI.

### Six stages of the SROI method applied to Satellite programs

This protocol reports on the six stages of the SROI methodology (26) as summarised here:

1. Establishing scope and identifying and involving stakeholders.
2. Mapping outcomes.
3. Evidencing outcome and giving them a value.
4. Establishing impact.
5. Calculating the SROI.
6. Reporting and translating results to stakeholders.

Each of the six stages will be further detailed in reference to the programs offered by Satellite, refencing the principles where applicable. The SROI methodology is underpinned by eight principles of social value (32) which guide how decisions are made to create a consistent and credible account of value. The principles are:

1. Involve stakeholders.
2. Understand what changes.
3. Value the things that matter.
4. Only include what is material.
5. Do not overclaim.
6. Be transparent.
7. Verify the result.
8. Be responsive.

## Establishing scope and identifying stakeholders

The first step involves delineating clear boundaries about what the SROI analysis will cover, who will be involved in the process and how. Satellite’s Youth Advisory Council (YAC) was involved as key stakeholders in setting the parameters for the SROI. The YAC is the youth advisory body for Satellite, who use their lived and living experience to influence and shape the work of Satellite. They were also invited to assist in the SROI process. At the beginning of the process, a one-day workshop was conducted, which sought to elicit YAC feedback on the evaluation and SROI plan with a focus on the types of outcomes they valued from participating in Satellite’s programs, the questions we should be asking in interviews, and guidance on the selection of measurement instruments [see (30) for further information about this day]. In addition, a member of the research team meets every 2 to 3 months with the YAC throughout the course of the project to monitor progress and collaboratively review results. Fortnightly meetings are also held between Satellite management and the evaluation team to facilitate the SROI process, e.g., participant recruitment.

The targets for change as a result of participating in the programs under investigation are the children and young people attending three Satellite programs. It is acknowledged groups other than the children and young people may experience value as a result of being involved with Satellite, either independently or via the benefits experienced by the children.

Parents or guardians of participants are informants on children’s progress and outcomes but not involved as targets of change in the programs. Another stakeholder is governments which provide services that may experience reduced demand as a result of benefits experienced by the children and young people. Guided by the social value principles, all stakeholders who experience change—positive or negative, intended or unintended—should be considered in the analysis. Nonetheless, to keep the SROI feasible and replicable, it was decided by the research team and Satellite management that the SROI focus on children and young people only. By focusing exclusively on children and young people, the SROI analysis can accurately capture the specific benefits and outcomes relevant to this demographic (23). This focus also allows for direct engagement with the primary end users, (namely young people) whose perspectives and experiences can be systematically and exclusively included in the analysis. It is acknowledged that the omission of other stakeholders who may be experiencing outcomes from the SROI may result in a devaluing of the programs. The SROI focus on three representative, capstone programs including:

- Satellite Camp for 8-12-year olds.
- Create and Connect for 8-17-year olds.
- Satellite Connect for 18-25-year olds (see Table 1 for more detail).

**Table 1:** Satellite programs.

Programs	Participants’ ages (approx.)	Brief description
Satellite Camps	8–15	Satellite Camp is a three-day overnight program for young people with family members who experience mental health challenges, giving them the opportunity for respite from their caring role. It aims to improve young people’s confidence, develop new skills and establish meaningful connections with other young participants who come from similar families. There is a mix of engaging outdoor and indoor creative activities that create a fun and safe environment for building skills and confidence, while making peer connections with other young people living with similar families. Camp facilitators provide role modelling for adaptive coping and normalising conversations about mental illness.
Create and Connect	8–17	These workshops are one-day programs that offer opportunities for participants to connect with peers in a safe, creative space. Various workshops are offered including music and song writing, photography and visual art such as graffiti and meme-making, plus other art and craft-based activities. Participants learn how to use music, photography, and art to express themselves, in an environment where they have the opportunity to connect with other young people who have a family member living with mental health challenges. The workshop allows participants to explore their personal and shared experiences through open dialogue and develop inclusive communication skills, including both visual and spoken language.
Satellite Connect in person or online	18–25	A structured peer support and peer development program that gives young people the space to be heard, learn new skills, and share their stories. Also covered is how their lived experience might be used to positively shape and influence the lives of younger children. Online versions of Satellite Connect run for 6 weeks, while in-person editions of Satellite Connect takes place across 3 weeks. During the program, participants meet other young people who have a family member living with mental health challenges, and interact, collaborate, and form connections with others living with similar families. Various themes are discussed including self-care and self-compassion, communicating creatively, talking about mental health, mental illness, language and stigma, and considering next steps for participants to practice newly acquired skills.

These programs were selected because they provide a good overview of participants’ ages and represent the capstone programs offered to young carers. Given they attract the most participants, this focus also provides rich data for analysis. The SROI covers the period of January 2023 through to June/July, 2024 with the final evaluation report to be delivered in August 2024.

## Mapping outcomes

The second phase details how the resources (inputs) to deliver the activities being analysed (measured as outputs) result in valued outcomes for stakeholders (young people). The relationship between inputs, outputs and outcomes is sometimes called the Theory of Change, which essentially outlines how programs intend to make a difference to those impacted (26). The inputs include what Satellite and other funders contributes in terms of the various program offerings involving staff expertise and time, venue and other resources. The *activities* are the three programs outlined above.

To determine *outcomes,* we first invited the YAC to identify and then prioritise the positive outcomes they considered to be important, resulting from Satellite participation (see Table 2). Many of these preferences are very similar (i.e., around connectedness and belonging and the reverse, loneliness) and consequently are considered very important by the YAC (representing Satellite’s core population group - young people with family members who have mental health challenges). At this point, a Theory of Change was generated through consultation with Satellite staff and the YAC (see Figure 1). One of the key inputs included in the Theory of Change were the skills and backgrounds of the program facilitators. Satellite programs are facilitated by three staff with distinct roles and expertise in creativity (e.g., fostering self-reflection through creative activities) mental health (e.g., by facilitating conversations around mental health) and lived experience (providing a positive role model). This combination of facilitator input utilises the evidence-based benefits from embracing creativity (33) and embedding mental health promotion, prevention and early intervention within a lived experience lens. Other inputs include physical resources and funding, all of which are applicable to three of the programs included in the SROI. The same Theory of Change (inputs, and outcomes) relate to all listed programs (see Figure 1).

**Table 2:** YAC generated list of preferences for program outcomes.

Satellite outcomes	Net votes
Connectedness	9
Belonging	8
Reduced loneliness	7
Reduced shame	7
Help seeking	7
Acceptance of self and others	7
Understanding about mental health challenges	6
Improved coping	5

The Theory was also informed by an existing Satellite program logic model but condensed to include only children and young people and to consider only the three programs targeted in the SROI. Specific outcomes generated from the resulting Theory of Change include (i) a sense of belonging and increased connectedness (ii) increased resilience, and (iii) a sense of hope, optimism and possibility. We also needed to consider potentially negative outcomes to ensure that we provide a true and fair picture that allows Satellite to continuously improve and assess trade-offs between outcomes. Potentially negative outcomes will be identified in the interviews with young people.

## Evidencing outcomes and giving them a value

### Evidencing outcomes

This stage involves finding data that demonstrates whether preferred outcomes have occurred and then valuing those outcomes. A convergent parallel mixed method approach will be employed, consisting of qualitative and quantitative data collection and analysis and then comparing and relating the two before interpreting them (34).

All participants will be asked demographic questions about their age, gender identity, cultural background, language spoken at home and the nature of their family member’s illness. They will also be asked which Satellite program/s they have been involved in.

For the quantitative component of the evaluation, a within-subject pre-post study design will be used to identify potential changes over time (35). Accordingly, participants (and their parents, for children aged 18 years and under) in targeted programs will be invited to participate in questionnaires on program entry, and then 6 months later, using the number of programs as a covariate (as some will attend multiple programs). Participants are asked to record a nickname when completing questionnaires, to ensure traceability across time one and two (pre and post). Individual and longitudinal conversations will also be employed. Further detail for these steps is presented below.

### Measures

Measurement tools and processes were identified as per the program outcomes highlighted in the Theory of Change. In discussion with the YAC, it was determined that *belonging* referred to feelings of safety and comfort associated with particular groups of people. ***Connectedness*** was similar and referred to the quality of relationships (connections) with others, especially peers. It was on this basis that the Strengths and Difficulties Questionnaire [SDQ; (36)] was used as it provides a measure of ***prosocial behaviour*** (1 subscale, 5 items) in children, adolescents and young adults. The SDQ can be administered to parents or to the young person themselves if they are aged 11 years or over. Each item is answered based on a 3-point Likert scale (not true, somewhat true, certainly true) e.g., “I am helpful if someone is hurt, upset, or feeling ill.” Scores range from 0 to 10, with higher scores indicative of greater prosocial behaviour. The SDQ prosocial subscale has fair internal consistency (*α* = 0.75) and strong test–retest reliability over 12 months (*r* = 0.64; 66). Further, the SDQ subscales have age and gender norms specific to each country (including Australia), so that scores from the sample of interest can be compared with the same population from which they were drawn. The prosocial behaviour subscale will be used to measure ***connectedness*** as it examines the young person’s ability to relate well with peers. The Longitudinal Study of Australian Children (LSAC) items that pertain to connectedness will also be used to evidence this outcome (see below discussion on deadweight for an overview of the LSAC study).

***Resilience*** will be measured by the total score of the Children and Youth Resilience Measure – Revised [CYRM-R; (37)]. Resilience in this context is defined as “the capacity of individuals to navigate their way to the psychological, social, cultural, and physical resources that sustain their well-being, and their capacity individually and collectively to negotiate for these resources to be provided in culturally meaningful ways” [(38); p. 225]. The CYRM-R is a 17 item self-report measure of social-ecological resilience. Participants respond to the items using a 5-point Likert scale from *not at all* to *a lot*, e.g., “I know how to behave/act in different situations.” The CYRM-R has good internal consistency (*α* = 0.82) and a Rasch analysis indicated that both subscales have good ability to discriminate between people with varying levels of resilience (67).

Given the Theory of Change (Figure 1) which positions adaptive coping as an intermediate outcome leading to resilience, two coping measures will also be used to evidence resilience; the Kids Coping Scale [KCS, completed by children aged 10 years and under; (39)] and the Coping Across Situations Questionnaire [CASQ, completed by older children, adolescents and young adults; (40)]. The KCS has nine items with two subscales: emotion-focused and problem-focused coping. Children answer the items, e.g., “You avoided the problem or where it happened” using a three-point Likert scale (never, sometimes, a lot). The KCS has fair internal consistency (*α* = 0.30–0.58). It is acknowledged that the KCS has relatively low reliability. However, it should be noted that there is a lack of validated measures on coping for young children especially those who are at risk for their own mental health difficulties (41). A similar measure has been developed more recently, i.e., the Coping Questionnaire Child (42) which reports fair reliability (α = 0.68) and has fewer items. However, the measure involves children responding to vignettes relating to anxiety-provoking situations that are read to them by an examiner. We chose the KCS as it employed simple language, allowed children to complete the measure independently, and related to their general coping styles rather than specific anxiety-provoking situations. This is acknowledged to be a limitation of the project.

The CASQ has 20 items which measure coping strategies across three different areas: active (using social resources to solve problems), internal (appraising situations and searching for a compromise) and withdrawal (avoiding the situation) e.g., “I try to get help and comfort from people who are in a similar situation.” Young people rate each item on a 5 point Likert scale from *not used* to *always used*. The CASQ has fair to good internal consistency (*α* = 0.73–0.80) and moderate to strong test–retest reliability over 1 year (*r* = 0.47–0.88).

A sense of ***hope***, ***optimism and possibility*** will be measured using the Children’s Hope Scale [CHS; (43)]. The scale assesses whether children can identify a means to carry out goals (pathways) and their ability to initiate and sustain action towards goals [agency; (43)]. The measure is comprised of six self-report items rated on a 6-point Likert- scale (from not at all to all of the time) e.g., “Even when others want to quit, I know that I can find ways to solve the problem.” Scores can range from 6 to 36, with higher scores indicative of greater agency and goal attainment. The CHS has fair to good internal consistency (*α* = 0.72–0.86) and strong test re-test reliability over 1 month [*r* = 0.71; (43)].

### Conversations with young people

Individual ***conversations*** with young people/children will also be conducted as another way of evidencing all three outcomes. The YAC recommended calling the interviews “conversations” to be less formal and intimating and that is the language we use here also. Seven to nine young people from each of the identified programs will be invited to a conversation about their experiences of the programs, and self-perceived outcomes, both positive and negative. We anticipate up to one hour for these conversations and with parental and child consent they will be audio-recorded. Transcripts will be analysed within an inductive qualitative paradigm, using the six-step reflexive thematic process recommended by Braun and Clarke (44, 45) which involves becoming familiar with the data, generating initial codes, searching for and reviewing the themes, and then defining and naming the themes.

In addition, we will conduct ***longitudinal conversations***, at three time points with the same nine participants, over the duration of the project. Participants will be drawn from across the three identified programs. Given that many young people attend more than one program, these conversations will allow us to explore the cumulative impact of Satellite’s offerings, looking for instances of continuity, change and growth over time (46). Those who discontinue their relationship with Satellite will also be invited to be interviewed, to ascertain the reasons why they have disengaged, and what impacts may have occurred and remain (if any) from program participation. A within-case analysis will be conducted within each conversation data set, followed by a cross-case analysis of patterns that may occur over time and across conversations (46). All conversation schedules were developed with the YAC to ensure that the language was strength-based and age-appropriate and that content aligned with preferred program outcomes.

### Data analysis

The findings from both quantitative and qualitative components of the project will be integrated following separate analysis using a mixed methods approach adapted from Lieber’s (47) conceptual model. Qualitative interview data from the various stakeholder populations will initially be analysed thematically (44) while quantitative data will be analysed via traditional methods including repeated-measures MANCOVA. Following these two separate processes, the categorical dimensions of the qualitative themes will be developed, which are ‘grounded’ in the raw data (47) through a process of constant comparative analysis and robust discussion amongst the research team. These grounded dimensions will then be integrated with the results from the quantitative data, with the analysis process facilitating meaning making of the quantitative data that describes the factors and/or circumstances (e.g., age, program type and ‘dosage’, cultural background, gender) that may be associated with various outcomes, incorporating also stakeholders’ perspectives (the YAC and Satellite management) on why and how these outcomes were delivered.

#### Giving outcomes a value

In the final part of this process, we give the identified outcomes a monetary value using financial proxies that reflect that importance of the outcome to stakeholders (see Table 3). In consultation with the YAC, a sense of connectedness and sense of belonging was considered similar to having friends. Powedthavee (64) identified the economic value of making and having new friends, in addition to existing friends, and this value was subsequently updated and converted to Australian dollars (See Table 3). Given that the original value was calculated in 2007, we adjusted it for inflation to reflect its current purchasing power, ensuring consistency with contemporary economic conditions. Although using a currency converter may not be the ideal method, this approach, when combined with inflation adjustments, provides a reasonable approximation of the value in today’s terms.

**Table 3:** SROI stage 3 outcomes.

Stakeholder		Stage 3				
Satellite young person participants		The outcomes (what changes)				
	Outcome	How will we know if the outcome has occurred	Source	How can you value the change?	Value $AUD for six-month period	Source
		Measured how?	Where this information comes from	What proxy is used to value the change?	Value of the change	Where this information comes from
	Sense of belonging and increased connectedness	Feel more connected to other young people living in similar circumstances	SDQ: Prosocial behaviour subscale (child and parent versions) LSAC items on connectedness Interviews with children/youth	Powdthavee (64) identified the economic value of friends (in addition to existing friends): 15,500 pounds per year in 2007 is equivalent to 23,903.38 pounds in 2022, converted to 2022 AUD 42,818.76 *per annum*	$21,861.38	Powdthavee (64).
		Reduced feelings of isolation and increased feelings of belonging				
	Increased resilience	Decreased mental health crises	CYRM-R total score KCS, completed by children aged 10 years and under; CASQ, completed by older children, adolescents and young adults Interviews with children/youth	The Australian Psychological Society (APS) recommends $300 per hour per child/young person for psychological services – 14 sessions	$4,200 per child/young person	APS website: https://psychology.org.au/psychology/about-psychology/what-it-costs
	Sense of hope, optimism and possibility	Increased hope for the future that results in planning for future activities/goals, agency	Children’s Hope Scale Interviews with children/youth	Cost of a mentor such as what might be available from the Big Brothers, Big Sisters mentoring program: $2,500 per person for 12 months	$1,250	Big Brother, Big Sisters of Australia Annual Report 2019–2020 BBBS_Annual_Report_2019-2020.pdf (bigbrothersbigsisters.org.au)

We also identified a financial proxy for resilience. For the young people involved in these programs, resilience refers to achieving positive outcomes (inclusive of wellbeing) despite coming from challenging backgrounds, and adaptively coping with current stressors (see Figure 1, Theory of Change). According to Olsson et al. (48), promoting resilience at an individual level involves developing personal coping skills and resources, both of which they suggest may be obtained from one to one therapy. Pascual-Leone et al. (49) suggested 14 individual sessions is appropriate for individuals with existing issues. It was on this basis that resilience was valued as 14 individual, psychotherapeutic sessions (see Table 3). The assumption that 14 individual psychotherapeutic sessions has a similar effect to a one-day workshop (e.g., the Create and Connect program) is admittedly contestable. However, a one-day workshop that focuses on creativity and connectedness can offer an intensive, immersive experience that might be considered similar to the impact of multiple counselling sessions (33, 50). Moreover, the unique therapeutic mechanisms of creative programs can lead to profound insights and emotional processing (51–53), potentially achieving in 1 day what might take multiple counselling sessions. The added dimension of group dynamics in these programs which emphasises peer support, can amplify the overall therapeutic impact (33, 50, 51), again potentially contributing to outcomes comparable to multiple individual counselling sessions.

The final outcome involves an increased sense of hope, optimism and possibility, which has been equated to future planning and projecting oneself into the future (54). In identifying a comparable proxy, we are suggesting that the programs offered by Satellite are comparable to having a mentor. Mentors help with goal setting and guidance with planning to achieve those goals; mentors also help young people navigate challenges, make important decisions, and offer advice on various aspects of life (55). Thus, in valuing this outcome, we are equating having a mentor to participating in Satellite programs, which likewise aim to build young people’s hope, optimism and a sense of possibility.

## Establishing impact

This step examines aspects of change that would have happened anyway or are a result of other factors, both of which need to be considered and eliminated. There are four parts to this stage as outlined below.

### Deadweight and displacement

Deadweight identifies changes in participant circumstances or resources that might have occurred regardless of whether the program or activity had taken place. One of ways we will calculate deadweight, is be using data from the Longitudinal Study of Australian Children (LSAC) survey, as a benchmark or comparison group of young people not involved in Satellite’s programs. The LSAC includes a group of Australian children/adolescents who have self-identified as having caring responsibilities (56). These will be matched with some of the young people who have participated in Satellite’s programs on demographic variables of age, gender and socio-economic status. Commencing in 2003, the LSAC is a national longitudinal study of data collected every 2 years on various child, parental and family characteristics that influence children’s development at different ages (56). Specific LSAC items will be used, as pertaining to connectedness /belonging outcomes, though noting that comparative data are only available for young people aged 14 years and above. This means that only some of the Satellite group will be included, and only on the connectedness outcome, with children under 13 years of age not being compared to the LSAC group and other outcomes not being compared. It is possible that one or more young people from the LSAC cohort may have attended a Satellite program. Given that Satellite is a unique program only available to young carers living in Victoria (while LSAC is an Australian cohort), and the level of help-seeking within this cohort will be variable (collected as frequency data, not details of programs), the likelihood is considered small within the Victorian LSAC participants and unlikely for those residing interstate.

An *a priori* power analysis was conducted using G*Power version 3.1.9.7 software (65) to determine the minimum sample size required for the study. The required sample size to achieve 80% power for detecting a medium effect at a significance criterion of *α* = 0.05 will be 29 per group {intervention [Satellite participants] and usual care [LSAC]; as per (30)}. Other deadweight measures will be ascertained by asking young people what they perceive would have happened if they had not attended Satellite and their estimation of the impact of that occurrence (in the interviews). This is necessary as there is a risk that LSAC participants may be engaged in programs similar to Satellite. Their responses will be used to inform and qualify any differences between the LSAC calculations and the Satellite participant sample as well as deadweight across other age groups and for other outcome measures not included in the LSAC data set.

Displacement is an assessment of how much the outcome might have displaced other outcomes, including the occurrence of negative outcomes for the children and young people and others. An evaluation of a similar program {Paying Attention to Self [PATS]; (57)} found that building new connections with participants during the program came at the cost of building peer relationships in other sites outside the program such as school and this is a concern that could apply here. Alternatively, participants could be gaining transferrable social skills at Satellite that then lead to better connections at school and other settings. We will be investigating this and other potential outcomes in the conversations with young people and who they are turning to.

### Attribution

Attribution is a consideration of who else could have contributed to the outcomes, which helps to identify stakeholders and activities that can also play a role in change. This could be, for example, a young person who attended Satellite but also received sessions from a psychologist or took medication for their own mental health challenges. We are addressing attribution by controlling for children and young people who have participated in programs in the past at baseline, compared with those who are new to Satellite. This will account for the differences in their lived experience before starting their next/first program. We are also asking participants if they have accessed carer services and/or health professionals in the last 6 months at each time point, so that those who engage with more than one service and/or health professional can be accounted for as well.

### Duration and drop off

Duration refers to how long an outcome lasts for, while drop off acknowledges that outcomes may continue to last for many years but may decrease over time, or if it is sustained, may be influenced by other factors. To calculate drop off, we will deduct a fixed percentage from the remaining level of outcome at the end of 6 months. This decision will be based on the longitudinal interviews, which follow young people over the course of the project, including those who have maintained a relationship with Satellite and those who have not.

## Calculating the SROI

Calculating the SROI involves adding up the outcomes (value), applying the discounts (attribution, displacement, deadweight and drop off) and comparing the result to the investment or costs incurred in delivering the program. Costs involved for Satellite involve employing staff to facilitate programs (including administration and registration), insurance, venue hire, catering, materials and travel (Satellite sometimes pays for transport so that young people/children can attend programs). Expenses associated with reimbursing YAC members for their input and advice on the programs offered is also included. Our analysis spanned a six -month period only (as indicated in Table 3), which does not necessitate the use of a discount rate to account for the time value of money (58). As the timeframe is less than a year, no additional adjustments for the differing value of money over time were required.

Once the net present value of costs and outcomes have been established (taking into consideration drop off, deadweight and displacement), the final ratio can be calculated. The formula for calculating the return on investment is:

SROI ratio = Social value of stakeholder outcomes (discounted)

Cost of providing programs

According to Arvidson et al. (23), an SROI ratio greater than one indicates a positive return on investment, or in other words, where the benefits of the investment are greater than its costs. The calculation is based on proxy values, as outlined in Table 3.

As the results are influenced by non-quantitative variables and assumptions, a sensitivity analysis for the SROI will be calculated to promote robustness. After establishing a base case scenario as outlined above, each key assumption will be changed (by ±10%), one at a time, to ascertain how much the SROI ratio changes. Changes in the SROI will be compared for each variation and those assumptions that cause the most significant changes in the SROI ratio identified. Several plausible scenarios will be developed including best case, worse case and moderate scenarios, using most optimistic to most pessimistic values of each of the assumptions. Each of these scenarios will be reported including a discussion of which assumptions have the most significant impact on the SROI ratio.

## Reporting and translating results to stakeholders

This step involves sharing findings with stakeholders and responding to them, embedding good outcomes processes and verification of the report. We intend to share and workshop results with the YAC, Satellite management and funders. A research paper outlining the results will also be submitted as well as a video intended for public dissemination, highlighting the main SROI findings.

## Ethics statement

Ethical approval was obtained from the Monash Human Research Ethics Committee. A detailed information sheet and consent form will be provided to parents/carers of all potential child participants (under 18 years of age) along with an explanatory statement and assent form for children and youth. Written parental consent and child assent are both required for project engagement (for those with children under 18) while those young people who are older do not require parental consent. All relevant ethical principles will be adhered to, including privacy, confidentiality, and informed consent. Information will be distributed via email, text and in hard copy and Satellite staff will not be informed as to who is and is not involved. A member of the research team will be available to respond to questions about the study and to assist in completing the measures, if required.

## Limitations

A potential limitation inherent in any SROI study is the monetisation of outcomes (28) and a lack of a universal bank of indicators (59). However, even those things with a market value are valued subjectively based on market conditions and consumer preferences. It was challenging to identify financial proxies for the various outcomes in this study, especially in relation to resilience as it comprised of multiple factors. As pointed out by Mook et al. (60), the choice of financial proxies can become subjective, which can compromise the reliability of the SROI. Given these challenges, Nicholls et al. (26) highlighted the importance of being transparent about the development and identification of these proxies which this protocol aims to do. Nonetheless, we agree with the notion purported by Arvidson et al. [(23), p. 233] when they argued that excluding significant outcomes which may be challenging to place a monetary value on, would “render the analysis precisely wrong, rather than the desirable “roughly right” and would greatly diminish the perceived social value of a given program.

We acknowledge the relatively short-term nature of the study and our subsequent inability to track long term change (or lack thereof). The study would have been strengthened had we been able to locate a comparison group for children under 14 years of age and for other outcomes besides connectedness. Using a currency converter to update the 2007 value of friendships presents a limitation in our analysis and may not fully capture more nuanced economic changes over time. We also acknowledge that the decision to aggregate the number of programs without employing a weighting system may limit the analysis. Similarly, the various programs offered by Satellite are delivered by different facilitators. As the knowledge, experience, skill and interpersonal style of group facilitators can have a significant impact on program outcomes (61), it will be difficult to determine which specific program/s are responsible for participant outcomes. Nonetheless, we are collecting fidelity logbooks to document the consistency of program delivery (30).

## Conclusion

Given the financial constraints and challenges governments face when deciding between the allocation of limited resources, it is critical that methods to calculate the Social Return on Investment are transparent. This protocol contributes to an understanding of how the social value of programs for young people in families living with mental health challenges can be ascertained, by outlining the financial proxies for program outcomes. It also highlights the social value that comes from the nature of various programs with, and for, a population of young people who face myriad socioeconomic disadvantages that are often overlooked. If results are positive, conducting studies such as this will provide further evidence for the claim that community programs, such as those offered by Satellite, provide value for money by preventing more costly mental health and social interventions in the longer term (62).

Half of all lifetime cases of mental disorder start by the age of 14 years and three quarters by the age of 24 years (63) with other research demonstrating young people who have a family member who experiences mental health challenges, for a variety of factors, are at risk of developing poor mental and physical health outcomes (2). It is incumbent on governments to implement high quality early interventions and targeted supports that meet the specific needs of this group of young people. This protocol paper shows how initiatives such as those offered by Satellite might reduce costs associated with mental health crises in the future. The social impact on parents and/or other family members might be further explored in future studies.

The SROI may provide important findings that can be used to lobby for funding from government officials and philanthropists. Nonetheless, how and whether the SROI process and findings may be used to inform Satellite’s (and other similar organisation’s) business planning and contract negotiations has yet to be realised. Overall, the SROI methods may promote equitable resource allocation and ultimately, a better understanding of the broad implications of various social initiatives designed for this particular group of young people and children who are often disadvantaged by the social and economic systems and structures within which they and their families live.

## References

[1] Becvar RJ Becvar DS Reif LV. Systems theory and family therapy. 4th ed. London: Rowman & Littlefield (2024).

[2] Reupert A Maybery D Nicholson J Gopfert M Seeman M. Parental psychiatric disorder: Distressed parents and their families. Cambridge UK: Cambridge University Press (2015).

[3] Aldridge J. Where are we now? Twenty-five years of research, policy and practice on young carers. Crit Soc Policy. (2018) 38:155–65. doi: 10.1177/0261018317724525

[4] Zeighami R Oskouie F Joolaee S. The positive effects of parents’ mental illness on their children: a qualitative study. Bangladesh J Medical Sci. (2014) 13:449–53. doi: 10.3329/bjms.v13i4.12989

[5] Krzeczkowski JE Wade TJ Andrade BF Browne D Yalcinoz-Ucan B Riazi NA . Examining the mental health of siblings of children with a mental disorder: a scoping review protocol. PLoS One. (2022) 17:e0274135. doi: 10.1371/journal.pone.0274135, PMID: 36108083 PMC9477329

[6] Mowbray CT Bybee D Oyserman D Allen-Meares P MacFarlane P Hart-Johnson T. Diversity of outcomes among adolescent children of mothers with mental illness. J Emot Behav Disord. (2004) 12:206–21. doi: 10.1177/10634266040120040201

[7] Yates S Gladstone B Foster K Silven A Reupert A O’Dea L . Epistemic injustice in experiences of young people with parents with mental health challenges. Sociol Health Illn. (2024) 46:702–21. doi: 10.1111/1467-9566.13730, PMID: 37994180

[8] Goodyear M Cuff R Maybery D Reupert A. CHAMPS: a peer support program for children of parents with a mental illness. Australian e-J Advancement of Mental Health. (2009) 8:296–304. doi: 10.5172/jamh.8.3.296

[9] Gatsou L Yates S Goodrich N Pearson D. The challenges presented by parental mental illness and the potential of a whole-family intervention to improve outcomes for families. Child Fam Soc Work. (2015) 22:388–97. doi: 10.1111/cfs.12254

[10] Maybery D Reupert A Bartholomew C Cuff R Duncan Z McAuliffe C . An online intervention for 18-25-year-old youth whose parents have a mental illness and/or substance use disorder: a pilot randomised controlled trial. Early Interv Psychiatry. (2022) 16:1249–58. doi: 10.1111/eip.13274, PMID: 35118795 PMC9790290

[11] Foster K McPhee I Fethney J McCloughen A. Outcomes of theON FIREpeer support programme for children and adolescents in families with mental health problems. Child and Family Social Work. (2016) 21:295–306. doi: 10.1111/cfs.12143

[12] Reupert A Bee P Hosman C van Doesum K Drost LM Falkov A . Editorial perspective: Prato research collaborative for change in parent and child mental health – principles and recommendations for working with children and parents living with parental mental illness. J Child Psychol Psychiatry. (2022) 63:350–3. doi: 10.1111/jcpp.13521, PMID: 34582039 PMC9293418

[13] Reupert A Cuff R Drost L Foster K van Doesum K van Santvoort F. Intervention programs for children whose parents have a mental illness: a review. Med J Aust. (2012) 1:18–22. doi: 10.5694/mjao11.11145 25369843

[14] Reupert A Maybery D. “Knowledge is power”: educating children about their parent’s mental illness. Soc Work Health Care. (2010) 49:630–46. doi: 10.1080/00981380903364791, PMID: 20711943

[15] Marston N Stavnes K van Loon L Drost L Maybery D Mosek A . A content analysis of intervention key elements and assessments (IKEA): What’s in the black box in the interventions directed to families where a parent has a mental illness? Child Youth Serv. (2016) 37:112–28. doi: 10.1080/0145935X.2016.1104041

[16] Lannes A Bui E Arnaud C Raynaud J-P Revet A. Preventive interventions in offspring of parents with mental illness: a systematic review and meta-analysis of randomized controlled trials. Psychol Med. (2021) 51:2321–36. doi: 10.1017/S0033291721003366, PMID: 34435556

[17] Puchol-Martínez I Vallina Férnandez Ó Santed-Germán MA. Preventive interventions for children and adolescents of parents with mental illness: a systematic review. Clin Psychol Psychother. (2023) 30:979–97. doi: 10.1002/cpp.2850 36997159

[18] Wansink HJ Drost R Paulus A Ruwaard D Hosman C Janssens J . Cost-effectiveness of preventive case management for parents with a mental illness: a randomized controlled trial from three economic perspectives. BMC Health Serv Res. (2016) 16:228. doi: 10.1186/s12913-016-1498-z, PMID: 27388373 PMC4937554

[19] Creswell C Violato M Cruddace S Gerry S Murray L Shafran R . A randomised controlled trial of treatments of childhood anxiety disorder in the context of maternal anxiety disorder: clinical and cost-effectiveness outcomes. J Child Psychol Psychiatry. (2020) 61:62–76. doi: 10.1111/jcpp.13089, PMID: 31364169 PMC6916180

[20] Waldmann T Schaible J Stiawa M Becker T Wegscheider K Adema B . The cost-utility of an intervention for children and adolescents with a parent having a mental illness in the framework of the German health and social care system: a health economic evaluation of a randomized controlled trial. Child Adolesc Psychiatry Ment Health. (2023) 17:141. doi: 10.1186/s13034-023-00693-w, PMID: 38129868 PMC10740235

[21] Corvo L Pastore L Mastrodascio M Cepiku D. The social return on investment model: a systematic literature review. Meditari Accountancy Res. (2022) 30:49–86. doi: 10.1108/MEDAR-05-2021-1307

[22] Reupert A Maybery D Bartholomew C Cuff R Foster K Matar J . The acceptability and effectiveness of an online intervention for youth with parents with a mental illness and/or substance use issue. J Adolesc Health. (2020) 66:551–8. doi: 10.1016/j.jadohealth.2019.11.309 32001142

[23] Arvidson M Lyon F McKay S Moro D. Valuing the social? The nature and controversies of measuring social return on investment (SROI). Voluntary Sector Rev. (2013) 4:3–18. doi: 10.1332/204080513X661554

[24] Nicholls A Emerson J. Social finance: capitalizing social impact In: Nicholls A Paton R Emerson J, editors. Social finance. Oxford: Oxford University Press (2015). 1–42.

[25] McGrath R Stevens K. Forecasting the social return on investment associated with children’s participation in circus-arts training on their mental health and wellbeing. Int J Sociol Leis. (2019) 2:163–93. doi: 10.1007/s41978-019-00036-0

[26] Nicholls J Lawlor E Neitzert E Goodspeed T. A guide to social return on investment. 2nd ed. London: The Cabinet Office (2012).

[27] Hartfiel N Gittins H Morrison V Wynne-Jones S Dandy N Edwards RT. Social return on investment of nature-based activities for adults with mental wellbeing challenges. IJERPH. (2023) 20:6500. doi: 10.3390/ijerph20156500, PMID: 37569040 PMC10418598

[28] Arvidson M. Lyon F. McKay S. Moro D. (2010). The ambitions and challenges of SROI. Third sector research Centre. Retrieved from https://bigpushforward.net/wp-content/uploads/2011/09/the_ambitions_and_challenges_of_sroi.pdf (Accessed on 17th November, 2023)

[29] Daly S. Philanthropy, the big society and emerging philanthropic relationships in the UK. Public Manag Rev. (2011) 13:1077–94. doi: 10.1080/14719037.2011.619063

[30] Reupert A Freeman N Hine R Lea S Nandakumar N O’Grady C . Evaluating programs for young people with a family member with mental health challenges: protocol for a mixed methods, longitudinal, collaborative evaluation. BMC Psychol. (2023) 11:67. doi: 10.1186/s40359-023-01104-7, PMID: 36899413 PMC10000348

[31] Al-Jundi A Sakka S. Protocol writing in clinical research. J Clin Diagn Res. (2016) 10:ZE10–3. doi: 10.7860/JCDR/2016/21426.8865, PMID: 28050522 PMC5198475

[32] Social Value International. The principles of social value. (2021) Retrieved from https://static1.squarespace.com/static/60dc51e3c58aef413ae5c975/t/6127b55936e97e03e86297ea/1629992289441/Principles+of+Social+Value+.pdf (Accessed on 13th March, 2024)

[33] Riley S. Contemporary art therapy with adolescents. London: Jessica Kingsley Publishers (1999).

[34] Creswell J Plano-Clark V. Designing and conducting mixed methods research. 2nd ed. London: SAGE (2011).

[35] O'Connell NS Dai L Jiang Y Speiser JL Ward R Wei W . Methods for analysis of pre-post data in clinical research: a comparison of five common methods. J Biometrics and Biostatistics. (2017) 8:1–8. doi: 10.4172/2155-6180.1000334, PMID: 30555734 PMC6290914

[36] Goodman R. The strengths and difficulties questionnaire: a research note. J Child Psychol Psychiatry. (1997) 38:581–6. doi: 10.1111/j.1469-7610.1997.tb01545.x 9255702

[37] Ungar M Liebenberg L. Assessing resilience across cultures using mixed methods: construction of the child and youth resilience measure. J Mixed Methods Res. (2011) 5:126–49. doi: 10.1177/1558689811400607

[38] Ungar M. Resilience across cultures. Br J Soc Work. (2006) 38:218–35. doi: 10.1093/bjsw/bcl343

[39] Maybery D Steer S Reupert A Goodyear M. The kids coping scale. Stress Health. (2009) 25:31–40. doi: 10.1002/smi.1228

[40] Seiffge-Krenke I. Stress, coping and relationships in adolescence. New York: Taylor & Francis (1995).

[41] Jacobs P Power L Davidson G Devaney J McCartan C McCusker P . A scoping review of mental health and wellbeing outcome measures for children and young people: implications for children in out-of-home care. J Child Adolesc Trauma. (2024) 17:159–85. doi: 10.1007/s40653-023-00566-6, PMID: 38938951 PMC11199430

[42] Crane ME Kendall PC. Psychometric evaluation of the child and parent versions of the coping questionnaire. Child Psychiatry Hum Dev. (2020) 51:709–20. doi: 10.1007/s10578-020-00975-w, PMID: 32157488 PMC7483227

[43] Snyder CR Hoza B Pelham WE Rapoff M Ware L Danovsky M . The development and validation of the children’s Hope scale. J Pediatr Psychol. (1997) 22:399–421. doi: 10.1093/jpepsy/22.3.399 9212556

[44] Braun V Clarke V. Thematic analysis: A practical guide. London: Sage (2021).

[45] Braun V Clarke V. Using thematic analysis in psychology. Qual Res Psychol. (2006) 3:77–101. doi: 10.1191/1478088706qp063oa

[46] Foster K Mitchell R Van C Young A McCloughen A Curtis K. Resilient, recovering, distressed: a longitudinal qualitative study of parent psychosocial trajectories following child critical injury. Injury. (2019) 50:1605–11. doi: 10.1016/j.injury.2019.05.003 31101410

[47] Lieber E. Mixing qualitative and quantitative methods: insights into design and analysis issues. J Ethnographic & Qualitative Res. (2009) 3:218–27.

[48] Olsson CA Bond L Burns JM Vella-Brodrick DA Sawyer SM. Adolescent resilience: a concept analysis. J Adolesc. (2003) 26:1–11. doi: 10.1016/S0140-1971(02)00118-5 12550818

[49] Pascual-Leone A Yeryomenko N Sawashima T Warwar S. Building emotional resilience over 14 sessions of emotion focused therapy: Micro-longitudinal analyses of productive emotional patterns. Psychother Res. (2019) 29:171–85. doi: 10.1080/10503307.2017.1315779, PMID: 28468535

[50] Malchiodi CA. Art therapy and health care. New York: Guilford Press (2013).

[51] De Witte M Orkibi H Zarate R Karkou V Sajnani N Malhotra B . From therapeutic factors to mechanisms of change in the creative arts therapies: a scoping review. Front Psychol. (2021) 12:678397. doi: 10.3389/fpsyg.2021.678397, PMID: 34366998 PMC8336579

[52] Moula Z. “I didn’t know I have the capacity to be creative”: Children’s experiences of how creativity promoted their sense of wellbeing. A pilot randomised controlled study in school art therapies. Public Health. (2021) 197:19–25. doi: 10.1016/j.puhe.2021.06.004, PMID: 34274622

[53] Zarobe L Bungay H. The role of arts activities in developing resilience and mental wellbeing in children and young people a rapid review of the literature. Perspect Public Health. (2017) 137:337–47. doi: 10.1177/1757913917712283, PMID: 28613107

[54] Bryant FB Harrison PR. Measures of Hope and optimism In: Boyle GJ Saklofske DH Matthews G, editors. Measures of personality and social psychological constructs. London: Elsevier (2015). 47–73.

[55] Lakind D Atkins M Eddy JM. Youth mentoring relationships in context: mentor perceptions of youth, environment and the mentor role. Child Youth Serv Rev. (2015) 53:52–60. doi: 10.1016/j.childyouth.2015.03.007, PMID: 25866427 PMC4387543

[56] Sanson A Johnstone R. Growing up in Australia takes its first steps. Fam Matters. (2004) 67:46–53.

[57] Hargreaves J Bond L O’Brien M Forer D Davies L. The PATS peer support program: prevention/early intervention for adolescents who have a parent with a mental illness. Youth Stud Australia. (2008) 27:43–51.

[58] Lawler E. Neitzert E. Nicholls J. (2008). Measuring value: a guide to social return on investment (SROI), (2^nd^ ed.). New economics foundation. Retrieved from https://commdev.org/wp-content/uploads/pdf/publications/Measuring-Value-A-Guide-to-Social-Return-on-Investment.pdf (Accessed on 13th March, 2024)

[59] Bridgeman J Murdock A Maple P Townley C Graham J. Putting a value on young people's journey into construction: introducing SROI at construction youth trust In: Raidén AB Aboagye-Nimo E, editors. Procs 31^st^ annual ARCOM conference, 7–9 September 2015. Lincoln, UK: Association of Researchers in Construction Management (2015). 207–16.

[60] Mook L Maliorano J Ryan S Armstrong A Quarter J. Turning social return on investment on its head. Nonprofit Manag Leadersh. (2015) 26:229–46. doi: 10.1002/nml.21184

[61] Raine L Trammel RC. Experiential learning in generalist groups courses: skills and process of BSW and MSW students. Soc Work Groups. (2023) 47:317–36. doi: 10.1080/01609513.2023.2275672

[62] Reupert A. ‘Boots’ theory: why mental health initiatives need to address economic inequalities. Adv Ment Health. (2023) 21:85–7. doi: 10.1080/18387357.2023.2221553

[63] Kessler RC Berglund P Demler O Jin R Merikangas KR Walters EE. Lifetime prevalence and age-of-onset distributions of DSM-IV disorders in the National Comorbidity Survey Replication. Arch Gen Psychiatry. (2005) 62:593–602. doi: 10.1001/archpsyc.62.6.593, PMID: 15939837

[64] Powdthavee N. Putting a price tag on friends, relatives, and neighbours: using surveys of life satisfaction to value social relationships. J Socio-Econ. (2008) 37:1459–80. doi: 10.1016/j.socec.2007.04.004

[65] Faul F Erdfelder E Buchner A Lang A-G. Statistical power analyses using G*Power 3.1: Tests for correlation and regression analyses. Behavior Research Methods (2009) 41:1149–60. doi: 10.3758/BRM.41.4.1149 19897823

[66] Hawes DJ Dadds MR. Australian data and psychometric properties of the Strengths and Difficulties Questionnaire. Aust N Z J Psychiatry. (2004) 38:644–51. doi: 10.1080/j.1440-1614.2004.01427.x 15298588

[67] Jefferies P McGarrigle L Ungar M. The CYRM-R: A Rasch-Validated Revision of the Child and Youth Resilience Measure. J Evid Based Soc Work (2019) 16:70–92. doi: 10.1080/23761407.2018.1548403 30472932

